# Optimization of a Portable Adenosine Triphosphate Bioluminescence Assay Coupled with a Receiver Operating Characteristic Model to Assess Bioaerosol Concentrations on Site

**DOI:** 10.3390/microorganisms8070975

**Published:** 2020-06-29

**Authors:** Chun-Chieh Tseng, Yi-Chian Lu, Kai-Chih Chang, Chien-Che Hung

**Affiliations:** 1 Department and Graduate Institute of Public Health, Tzu Chi University, Hualien 97004, Taiwan; luyc1108@gmail.com (Y.-C.L.); gavink23@gmail.com (C.-C.H.); 2 Department of Laboratory Medicine and Biotechnology, Tzu Chi University, Hualien 97004, Taiwan; kaichih@mail.tcu.edu.tw; 3 Institute of Medical Sciences, Tzu Chi University, Hualien 97004, Taiwan

**Keywords:** bioaerosol, ATP, Coriolis µ air sampler, BioSampler, indoor air, benchmark, receiver-operating characteristic curve

## Abstract

Rapid monitoring of the microbial content in indoor air is an important issue. In this study, we develop a method for applying a Coriolis sampler coupled with a portable ATP luminometer for characterization of the collection efficiency of bioaerosol samplers and then test this approach in field applications. The biological collection efficiencies of the Coriolis sampler and a BioSampler for collecting four different types of bioaerosols, including *Escherichia coli*, *Staphylococcus aureus*, *Candida famata* and endospores of *Bacillus subtilis*, were compared in a chamber study. The results showed that the ATP assay may indicate the four microbes’ viability, and that their defined viabilities were positively correlated with their culturability. In addition, the optimal sampling conditions of the Coriolis sampler were a 200 L/min flow rate and a sampling time of 30 min. Under these conditions, there was no significant difference in sampling performance between the BioSampler and Coriolis sampler. In field applications, the best ATP benchmark that corresponded to culturable levels of < 500 CFU/m^3^ was 287 RLUs (sensitivity: 100%; specificity: 80%) for bacteria and 370 RLUs (sensitivity: 79%; specificity: 82%) for fungi according to receiver operating characteristic curve analysis. Consequently, an ATP criterion is recommended for indicating whether the corresponding airborne culturable concentrations of microbes meet those of published guidelines.

## 1. Introduction

The many different microbes in indoor air are referred to as bioaerosols and may pose health threats to humans by inhalation [1]. To prevent the transmission of bioaerosols, efficient methods for their rapidly detection are urgently required. Traditionally, culture assays are the most widely used method for bioaerosol detection. However, some disadvantages of these assays are that they are time-consuming and species-specific and take 1–3 days for colony formation [2]. In addition, most microbes in the viable but nonculturable (VBNC) stage cannot be detected by culture methods [3]. To overcome the disadvantages of culture-based methods, several nonculture approaches, such as epifluorescence microscopy, flow cytometry, PCR, and NanoGene assays, have already been applied to bioaerosol detection [4,5,6,7,8]. Some nucleic acid assays with high sensitivity and specificity can provide quick results but may not provide the viability of bioaerosols unless the method has been modified [8,9].

Among all non-culture methods, the adenosine triphosphate (ATP) bioluminescence assay has the potential for rapidly monitoring bioaerosols since ATP is present in all living organisms. In addition, the strength of the ATP bioluminescence signal is related to the microbe’s physiological conditions and is proportional to the bioaerosol concentration [10]. Consequently, ATP bioluminescence could be used as an indicator to assess the total microbe loads in indoor air. Implementing the use of ATP bioluminescence to monitor bioaerosols can be divided into two aspects: one is the development of a continuous and near real-time bioaerosol monitoring system [11,12,13,14,15], and the other is using ATP bioluminescence as a tool to evaluate the sampling performance of different bioaerosol samplers [2,16]. Although a continuous ATP bioluminescence system can obtain near real-time total bioaerosol concentrations without manual procedures, it may require specialized or expensive equipment. On the other hand, such a sampler or system may still be under development and would therefore not be immediately available for application in indoor environments.

To date, hospital surface cleanliness can be determined by ATP levels expressed in relative light units (RLUs). A clean surface was defined based on benchmark values from 100 to 500 RLUs [17]. Because the ATP assay can obtain results within a short time, establishing an ATP benchmark value will also be helpful for bioaerosol measurements. Currently, the quantitative standards or guidelines for bioaerosols have been recommended by governmental agencies and private organizations [3]. These quantitative standards/guidelines were established by culture-based methods and expressed in colony-forming units (CFU)/m^3^. Consequently, it would be possible to immediately determine whether the total bioaerosol concentration is qualified on site by investigating the relationship between ATP and CFU values through a statistical model.

To determine the ATP level in bioaerosols, applying a liquid medium for collecting bioaerosols was preferred over using an agar impactor when the ATP bioluminescence assay was conducted [2,12,16,18]. The application of an impactor and subsequent swabbing of the agar surface to obtain ATP levels may underestimate the ATP levels of bioaerosols. However, regardless of the type of sampler used to collect bioaerosols, the impact on microbial viability, including its assessment via ATP levels, during the sampling process still requires further assessment. At present, there are two types of ATP bioluminescence luminometers, portable and desktop. To examine the hygiene conditions of a surface, portable luminometers have been applied in hospital settings [19] and the food industry [20]. Although portable luminometers have good mobility, they are rarely used in bioaerosol monitoring. The performance of these portable instruments for bioaerosol quantification also requires investigation.

The aim of this investigation was to conduct a two-phase study of the application of a portable ATP luminometer coupled with a high-volume sampler for rapidly assessing indoor bioaerosol concentrations. In phase one of the study, a Coriolis µ air sampler (Coriolis sampler) was chosen since it has a high sampling volume (up to 300 L/min), which may reduce the sampling time. To our knowledge, the ATP bioluminescence assay has not been coupled with this sampler for detecting bioaerosols. Consequently, we evaluated the optimal conditions of the Coriolis sampler for the recovery of four different bioaerosols (*Escherichia coli*, *Bacillus subtilis* endospores, *Staphylococcus aureus* and *Candida famata*) by culture and ATP bioluminescence assays in a chamber. The traditional sampler BioSampler was used for comparison in phase one of the study. In phase two of the study, the Coriolis sampler coupled with culture and ATP assays under optimal conditions were applied in field environments, including hospitals, libraries and long-term care institutions. Finally, the environmental results obtained during phase two were all merged to identify a criterion that could indicate whether the culturable bioaerosols met a particular threshold on site based on different established guidelines or regulations by using receiver operating characteristic curve analysis.

## 2. Material and Methods

### 2.1. Phase One: Optimal Conditions for the Coriolis Sampler Coupled with the ATP Assay

#### 2.1.1. Test Microorganisms and Culture

The microorganisms used in this study were all purchased from the Bioresource Collection and Research Center (Hsin-Chu, Taiwan). Vegetative cells of *E. coli* (BCRC 10675), *S. aureus* (BCRC 11863) *and C. famata* (BCRC 22304) and the endospores of *B. subtilis* (BCRC 12145) were used in this study. These microorganisms represented gram-negative (*E. coli*) and gram-positive (*S. aureus*) bacterial cells. In addition, bacterial endospores (*B. subtilis*) and yeast cells (*C. famata*) were also evaluated to comprehensively represent the different types of bioaerosols in the environment. The *E. coli* and *S. aureus* strains were inoculated in Luria-Bertani (LB) broth (Difco, Detroit, MI, USA) and incubated for 14 h at 37 °C. After cultivation, the bacterial pellets were aseptically washed with phosphate-buffered saline (PBS) and transferred to a 15 mL conical centrifuge tube that was then capped and then centrifuged (500× *g*, 5 min). Finally, the supernatant was discarded, and the bacterial pellets were resuspended in sterile distilled water for preparation of spray suspensions.

For *B. subtilis*, the cells were initially inoculated on tryptone soy agar plates for cultivation at 37 °C for 7 days. After that time, the bacterial colonies were harvested into sterile distilled water and then shaken at 45 rpm for 24 h at room temperature. Subsequently, the samples were heated for 10 min at 80 °C to inactivate the vegetative cells. Finally, the resulting endospore suspension was centrifuged at 3500 rpm for 5 min. Under microscopic observation, the centrifuged *B. subtilis* suspensions were found to contain only endospores.

The cells of *C. famata* were inoculated in yeast malt (YM) broth (Difco) and incubated for 24 h at 25 °C. Similar to the preparation process of *E. coli* and *S. aureus*, the yeast pellets were washed with PBS, transferred to a centrifuge tube and then centrifuged (500× *g*, 5 min). Finally, the supernatant was discarded, and the pellets were resuspended in sterile water for the preparation of spray suspensions.

#### 2.1.2. ATP and Bacterial Standard Curves

The generation of standard curves was conducted with two goals: (a) to investigate the relationship between the portable luminometer signal (RLUs) and standardized ATP concentrations and (b) to determine the correlation between the ATP signal in RLUs and the culturable concentrations of four different types of microbes. To address the first goal, serial dilutions of a 1 mM ATP standard (adenosine-5-triphosphate disodium salt, Sigma-Aldrich, St. Louis, MO, USA) were prepared. Each serially diluted sample was brought into contact with a swab and incubated for 15 s. Then, the sample was placed into a portable luminometer (3M Clean-Trace ATP System; 3M Co., St. Paul, MN, USA) to measure the corresponding RLU value. To address the second goal, the four different microbes were serially diluted from 10^8^ to 10^3^ CFU/mL, and the RLU readings obtained from each sample were used to estimate the culturable concentration corresponding to the ATP readings.

#### 2.1.3. Preparation of Viable and Dead Microbes

To obtain dead (nonculturable) cells, bacterial and fungal cell suspensions of 10^5^ CFU/mL were heated at 85 °C for 10 min except for the endospores of *B. subtilis*, which were directly sterilized by an autoclave at 121 °C for 15 min. After heating, all microbes were no longer culturable on their corresponding test agar media. These dead microbes were defined as 0% controlled viable samples, whereas unheated samples were defined as 100% controlled viable samples. Subsequently, three different controlled viable samples (75%, 50%, and 25%) were prepared from the 0% and 100% samples. For each of the five samples (100%, 75%, 50%, 25% and 0%) of the different microbes, the culturable ratio was the average number of CFU from triplicate platings divided by that of the 100% controlled viable sample. In addition, the culturable and viable ratios of microbial cells for each of the five samples were determined by dividing the culture and ATP bioluminescence assay values by those of the 100% controlled viable sample.

#### 2.1.4. Aerosol Preparation and Test System

The aerosol generation system was similar to that of our previous study [21]. Four different bioaerosols were generated by a Collison three-jet nebulizer (BGI Collison Nebulizer, BGI Inc., Waltham, MA, USA) at 3 L/min into a test chamber with a volume of 97 L. The four bioaerosols at 10^8^ CFU/mL were coupled with an entrance air stream (flow rate = 300 L/min) toward the test chamber inlet. In our test chamber, the concentrations of the four bioaerosols were stable for at least 90 min (CV% = 10%). To simulate a moderate relative humidity (RH) of 55% in an indoor environment, a water vapor stream was adjusted by changing the flow rate ratio of humidified and dry gas streams. The actual RH in the test chamber was monitored with a hygrometer (Rotronic AG, Bassersdorf, Switzerland).

#### 2.1.5. Test Sampler and Sample Processing

**Figure 1:**
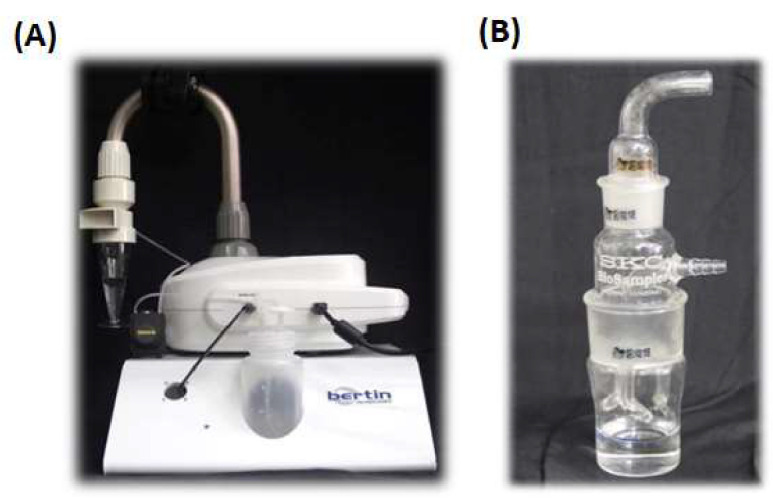
The sampling devices used in this study: (**A**) a Coriolis µ air sampler and (**B**) a BioSampler.

In this study, the ATP and culturable concentrations in the collected liquid samples were evaluated by a Coriolis sampler (catalog number: P001080-CORM0-A; Bertin Technologies, Montigny-le-Bretonneux, France) and a BioSampler (catalog number: 225-9595; SKC Inc., Connellsville, PA, USA) (Figure 1). In this test, the sampling flow rates of the Coriolis sampler were 100, 200 and 300 L/min, and different sampling times were also evaluated (5, 10, 30 and 60 min). The Coriolis sampler was filled with 15 mL of sterile deionized water to collect bioaerosols. Due to the high sampling flow rate of the Coriolis sampler, the collection liquid would be rapidly evaporated when accompanied by relatively long sampling times. Consequently, we used an autocompensation system in combination with the Coriolis sampler; in this way, the lost liquid was recorded and further automatically replenished. In addition, the sampling flow rate of the BioSampler was 12.5 L/min, and the sampling time was the same as that of the Coriolis sampler. The BioSampler was filled with 20 mL of deionized water to collect bioaerosols.

After sampling, the remaining volumes of deionized water from both samplers were measured. Subsequently, the deionized water from the Coriolis sampler and BioSampler was divided into two parts. The first part was inoculated on different agar media and at varied temperatures as described in Section 2.1.1. The other sample was subjected to ATP bioluminescence analysis; a liquid sample (5 mL) was separated from the collection medium, brought into contact with a sampling swab and then placed into a portable luminometer to obtain the RLU reading. Finally, airborne microbe counts and ATP levels in the two bioaerosol samplers were calculated based on the sampling flow rate, sampling time, plated volume and dilution ratio.

#### 2.1.6. Calculation of VR and CR Values for Air Sampling of Bioaerosols

Our study investigated the determination of microbe viability according to the ATP concentration before and after air sampling. Consequently, we could also obtain an additional indicator, viability recovery (VR), to represent the most effective sampling methods for preserving microbe viability. VR was defined as follows:

(1) $$\mathrm{VR}=\frac{V_{\mathrm{sampler}}}{V_{\mathrm{nebulizer}}}$$

where V_sampler_ is the RLUs/m^3^ determined for ATP levels (i.e., the level of viable bioaerosols per cubic meter of air that passed through the sampler) and V_nebulizer_ is the RLUs/mL in the nebulizer that was determined for ATP levels (i.e., the level of viable microbes in the nebulizer liquid).

Culturability recovery (CR) is an indicator of microbe recovery in terms of culturability and was used in our previous study [9]. CR can be used to investigate which bioaerosol sampler or which sampling condition may result in less damage to the microbe’s culturability. The advantage of CR is that the initial culturability can be adjusted to determine the biological efficiency of a bioaerosol sampler. CR was defined as follows:

(2) $$\mathrm{CR}=\frac{C_{\mathrm{sampler}}}{C_{\mathrm{nebulizer}}}$$

where C_sampler_ is the CFU/m^3^ determined for an agar plate (i.e., the number of culturable bioaerosols per cubic meter of air that passed through the sampler) and C_nebulizer_ is the CFU/mL in the nebulizer that was determined for an agar plate (i.e., the number of culturable microbes in the nebulizer liquid).

#### 2.1.7. Statistical Analysis

We used the Kolmogorov-Smirnov test to determine whether the sample data were normally distributed. After this statistical analysis, the data were analyzed with nonparametric tests because the probability associated with the Kolmogorov-Smirnov test for normality was <0.05. Differences in the CR or VR values between the Coriolis sampler and the BioSampler were determined using the Mann-Whitney-Wilcoxon test. The CR or VR values obtained when bioaerosols collected with different sampling flow rates and sampling times for the two samplers were compared using the Kruskal-Wallis test, followed by Dunn’s multiple comparison test to determine statistically significant differences (*p* < 0.05). The same statistical method was also used to compare the loss rates of sampling liquid in the Coriolis sampler resulting from high sampling flow rates. The relationship between the ATP readings in RLUs and the culturable concentrations of different microbes was determined by Spearman’s rank correlation.

### 2.2. Phase Two: Field Sampling in Hospitals, Libraries and Long-Term Care Institutions

#### 2.2.1. Sampling Locations

We collected aerosol samples from two hospital halls, two libraries and two long-term care institutions (LCIs) in Taiwan. These locations were chosen for sampling because the environment of these three types of institutions had already been regulated in Taiwan. The sampling locations in the hospitals, libraries and LCIs were outpatient halls, reading areas and public halls, respectively.

#### 2.2.2. Field Aerosol Sampling

In all six sampling locations, aerosol samples were collected in triplicate using the Coriolis sampler because our preliminary study demonstrated that the Coriolis sampler had no significant difference in collecting performance from the BioSampler under specific sampling conditions. Since most recommended protocols for detecting culturable bioaerosols utilize different types of impactors [22,23,24], we also utilized a MAS 100 (Millipore, Billerica, MA, USA) to collect bioaerosols in parallel in the field to determine whether the airborne bacterial or fungal concentrations met a particular threshold. The two sampling apparatuses were placed approximately 1 m from a wall. In addition, sampling was conducted at 1.2 m above the floor, which was within the breathing zone of individuals at each location. To prevent interference from air flow, the sampling location was 3 m from square ceiling diffusers.

The Coriolis sampler was operated at a flow rate of 200 L/min for 30 min according to the optimized conditions determined in our chamber study. After sampling, the sample processing procedure applied in ATP and culture assays was the same as that used in the chamber study. For the MAS 100, the sampling flow rate was 100 L/min, and the sampling time was 7 min. In the field study, we followed the standard method proposed by the Environmental Protection Administration in Taiwan using tryptic soy agar (TSA) to culture the total bacterial aerosols (NIEA E301.15C) and used malt extract agar (MEA) for total fungal aerosols (NIEA E401.15C). The TSA plates evaluated in the field were placed in an incubator at 30 °C for 48 h, and the MEA plates were incubated at 25 °C for 4 days.

#### 2.2.3. Statistical Analysis

To compare the airborne concentrations of total bacteria and fungi among the six sampling sites, we used the Kruskal-Wallis test, followed by Dunn’s multiple comparison test to detect statistically significant differences (*p* < 0.05). The relationship between the RLU values and the culturable concentrations of different microbes in the field was examined by simple linear regression. In addition, receiver operating characteristic (ROC) curves, with the culture assays of the MAS 100 impactor samples as the standards, were generated to investigate how well the ATP assay could predict the corresponding culture levels. The optimal cutoff points of RLUs for predicting the levels in CFU/m^3^ in the impactor samples were determined using the Youden index with the optimal sensitivity (sen) and specificity (spe). The results obtained from all sampling locations of the field study were included in this analysis.

## 3. Results

### 3.1. ATP Standard Curves; Correlation of ATP Contents and RLU Values

**Figure 2:**
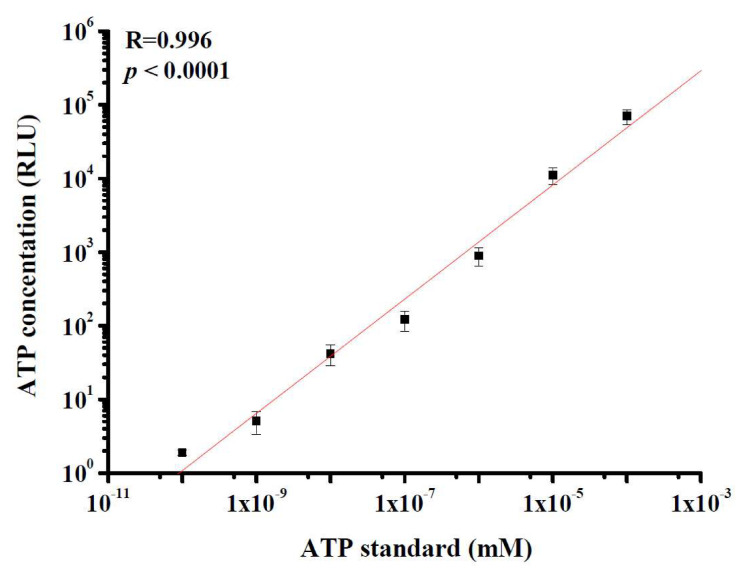
ATP standards ten-fold serially diluted in sterile water and their response in RLUs determined with a portable luminometer. Each error bar represents one standard deviation from the mean for triplicate experiments.

Figure 2 shows the portable ATP luminometer response to 10-fold serial dilutions of an ATP standard in sterile deionized water. The luminometer response linearly increased with ATP content, and the ATP content (mM) and RLU reading value were significantly (*p* < 0.0001) and strongly correlated (*R* = 0.996). The linear range of ATP content in this test was 10^−10^ to 10^−4^ mM. The detection limit of our portable ATP luminometers was 10^−10^ mM.

### 3.2. Culture Standard Curves; Correlation of RLU Values and Culture Results

**Figure 3:**
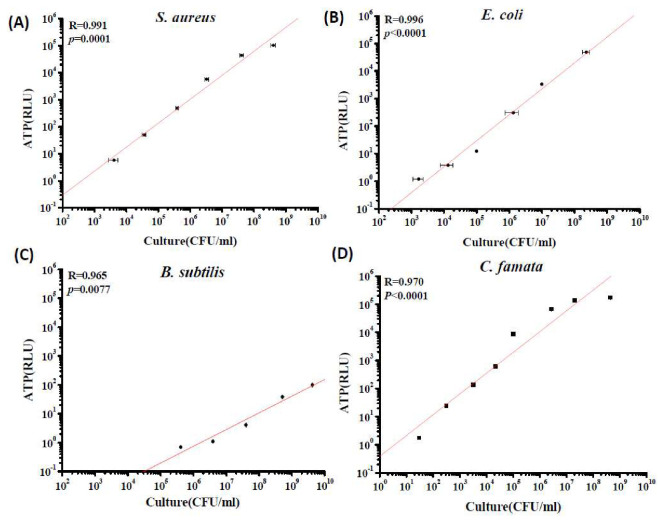
Serial dilutions of culturable (**A**) *S. aureus* cells, (**B**) *E. coli* cells, (**C**) *B. subtilis* endospores and (**D**) *C. famata* cells in sterile water and their response in RLUs determined with a portable luminometer. Each error bar represents one standard deviation from the mean for triplicate experiments.

Figure 3 shows the relationships between the colony counts of four different types of microbes at different concentrations and their corresponding RLU values in suspension. The colony counts linearly increased with increasing RLU values for all tested microbes in suspension. However, the slopes of the regression lines for both of the bacterial cell samples (0.99) were higher than those of the yeast (0.97) and endospore samples (0.96). The endospores of *B. subtilis* exhibited the lowest ATP content, and the culturable concentration of endospores had to be higher than 10^5^ CFU/mL to correspond to a reliable RLU value.

### 3.3. Comparison of Culturable and Viable Ratios as Measured by Culture and ATP Assays

**Figure 4:**
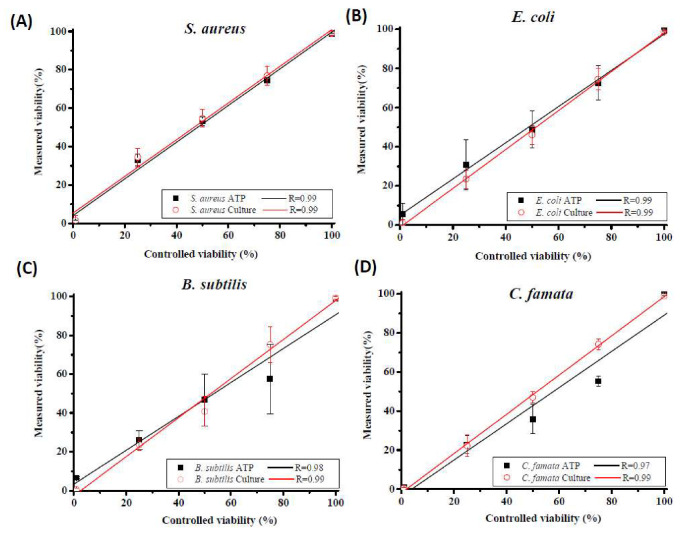
Correlations between the viability of controlled samples and their viability assessed using the culture and ATP bioluminescence assays. Three different controlled viable samples (75%, 50%, and 25%) were produced from 0% (heat-killed) and 100% (untreated) controlled viable samples. For each of the five controlled viable samples, the culturable (or viable) ratios of (**A**) *S. aureus* cells, (**B**) *E. coli* cells, (**C**) *B. subtilis* endospores and (**D**) *C. famata* cells were the average concentrations determined by the culture and ATP bioluminescence assays divided by that of the 100% controlled viable samples. Experiments were performed in triplicate, and the data are shown as the mean ± standard error of the mean.

The culturable or viable ratios of four different types of microbes measured using culture and ATP assays were compared. All culturable or viable ratios of the four different microbes measured using the culture and ATP assays were linearly related to the controlled viable samples with viabilities from 0% to 100% (Figure 4). The viable ratios measured by the ATP assay were not significantly different from the culturable ratios measured by the culture method (*p* = 0.90). According to Spearman’s rank correlation, the culturable ratios measured using the culture method were significantly correlated with the viable ratios measured using the ATP assay (*r* = 0.960; *p* < 0.001).

### 3.4. The Lost and Replenished Medium Volume during Air Sampling with the Coriolis Sampler

**Figure 5:**
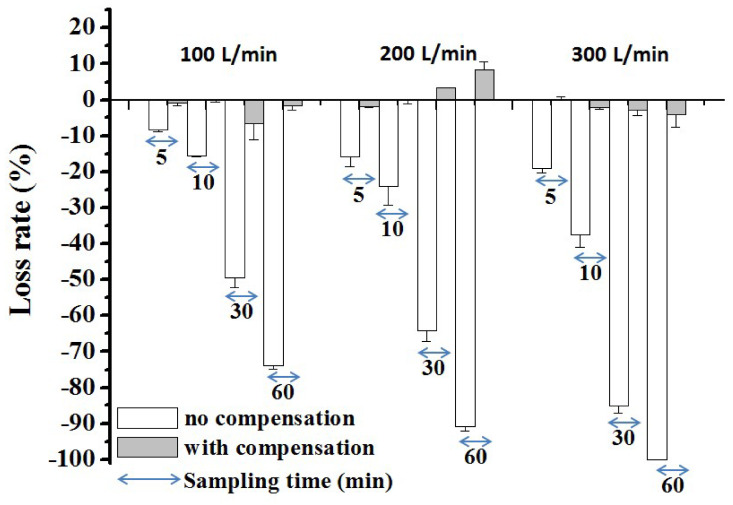
Loss rate of the collection liquid in the Coriolis sampler at different sampling flow rates and sampling times with/without a compensation system. Experiments were performed in triplicate, and the data are shown as the mean ± standard error of the mean.

Since the Coriolis sampler can be operated at high sampling flow rates (from 100 L/min to 300 L/min), the volume loss of the collection medium increased with increasing sampling volume. Without a compensation process at the lowest sampling flow rate of 100 L/min, only 8.3% of the collection medium was lost when the sampling time was 5 min (Figure 5). However, 73.8% of the sampling medium was lost if the longer sampling time of 60 min was implemented. With an increase in the sampling flow rate to 200 L/min or 300 L/min, 90.8% to 100% of the collection medium was lost after 60 min of sampling. By using an autocompensation system, the volume of replenished liquid could be adjusted by a microcomputer. Based on our preliminary tests, the replenishing flow rate of collection medium could be set to 0.1, 0.2 and 0.4 mL/min for sampling flow rates of 100, 200 and 300 L/min, respectively, to maintain a liquid loss (or increase) rate of less than 10%. This compensation system could maintain a stable collection medium volume for up to 60 min of sampling time regardless of the sampling flow rate.

### 3.5. VR and CR for Different Bioaerosol Species Collected by the BioSampler and the Coriolis Sampler

**Figure 6:**
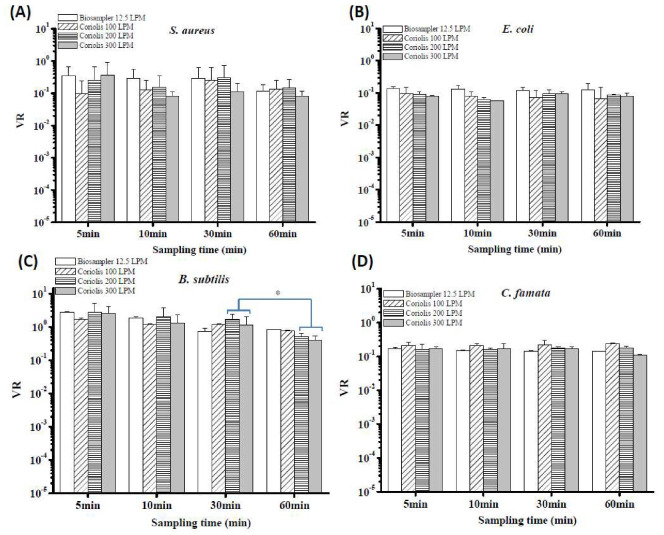
Relative viable collection efficiencies of the BioSampler and the Coriolis sampler for capturing airborne (**A**) *S. aureus* cells, (**B**) *E. coli* cells, (**C**) *B. subtilis* endospores and (**D**) *C. famata* cells. The indicator VR was used to adjust the initial viable concentration in the nebulizer to determine the biological performance of the two tested samplers. Samplers with high VR values can preserve ATP better than those with low VRs under the same sampling conditions. Experiments were performed in triplicate, and the data shown represent the mean ± standard error of the mean.

To compare the viable efficiency of the Coriolis sampler for collecting bioaerosols, the VR was investigated as an indicator through the ATP assay. For this test, a traditional BioSampler was used for comparison. In general, the VR values of *S. aureus*, *E. coli* and *C. famata* were not significantly changed with variation in sampling flow rates and sampling times (Figure 6A,B,D). The VR values of *B. subtilis* were also higher than those of the other three microbial strains (*p* = 0.04). In addition, at 200 and 300 L/min sampling flow rates, sampling times less than 30 min exhibited higher VR values than sampling for 60 min (Figure 6C; *p* = 0.05). Overall, there was no significant difference in VRs between the BioSampler and Coriolis samplers for collecting *S. aureus*, *B. subtilis* and *C. famata*. Nevertheless, the overall VR values of bioaerosols collected by the BioSampler were significantly higher than those collected by the Coriolis sampler for *E. coli* (*p* = 0.04).

**Figure 7:**
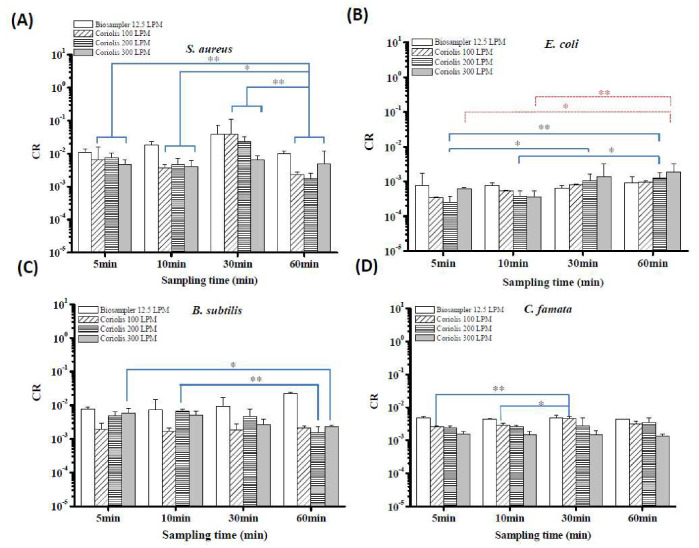
Relative culturable collection efficiencies of the BioSampler and the Coriolis sampler for capturing airborne (**A**) *S. aureus* cells, (**B**) *E. coli* cells, (**C**) *B. subtilis* endospores and (**D**) *C. famata* cells. The indicator CR was used to adjust the initial culturable concentration in the nebulizer to determine the biological performance of the two tested samplers. Samplers with high CR values can preserve culturability better than those with low CRs under the same sampling conditions. * *p* < 0.05 and ** *p* < 0.01 compared with the respective groups. Experiments were performed in triplicate, and the data shown represent the mean ± standard error of the mean.

To compare the culturable efficiencies of the Coriolis sampler for collecting bioaerosols, CR as an indicator was applied to adjust the initial culturability in the nebulizer. Figure 7 presents the measured CRs determined for the BioSampler and the Coriolis sampler for four different bioaerosol species at an RH of 55%. For *S. aureus*, the CR values of the BioSampler ranged from 0.01 to 0.04, and those of the Coriolis sampler ranged from 0.002 to 0.07 regardless of sampling flow rate (Figure 7A). There was no significant difference in CR values among the three different sampling flow rates of the Coriolis sampler. However, the CR values obtained from relatively short sampling times, including 5, 10 and 30 min, were significantly higher than those obtained for a sampling time of 60 min (*p* = 0.002, 0.028 and 0.000, respectively). Combining all the results in Figure 7A shows that the BioSampler had higher CRs than the Coriolis sampler for *S. aureus* collection (*p* < 0.001).

The CR values of *E. coli* collected by the Coriolis sampler at different sampling flow rates were not significantly different from each other (Figure 7B; *p* = 0.10). With relatively long sampling times (30 and 60 min), the CR values of *E. coli* were significantly higher than those for 5 and 10 min of sampling, respectively (Figure 7B). The CRs of the BioSampler were not significantly different from that of the Coriolis sampler at sampling times of 30 and 60 min (*p* = 0.12). However, the Coriolis sampler had significantly lower CRs than the BioSampler when operated for 5–10 min (*p* < 0.001).

For *B. subtilis* endospores, the CR values at 100 L/min were also significantly lower than those at 200 and 300 L/min (Figure 7C; *p* < 0.001). Different from the results for the two bacterial cell types, sampling for relatively short times, such as 5 and 10 min, yielded higher CR values for the *B. subtilis* endospores than sampling for 60 min at 200 and 300 L/min. Overall, the BioSampler performed better than the Coriolis sampler, achieving higher CRs for *B. subtilis* endospore collection (*p* < 0.001). Finally, the CR values of *C. famata* collected at 100 and 200 L/min were significantly higher than those at 300 L/min (Figure 7D; *p* < 0.001). At the lowest sampling flow rate of 100 L/min for the Coriolis sampler, sampling for 30 min yielded a significantly higher CR value than sampling for 5 and 10 min (*p* = 0.002 and 0.015, respectively). Among all results, there was no significant difference between the CR values for the Coriolis sampler and the BioSampler when *C. famata* were collected (*p* = 0.15).

The optimal conditions for the Coriolis sampler were determined using the efficiency indicators CR and VR and a statistical analysis of a post hoc comparison: a flow rate of 200 L/min combined with a sampling time of 30 min. Under these conditions, there was no significant difference in sampling performance between the BioSampler and the Coriolis sampler.

### 3.6. Applying the Coriolis Sampler Coupled with the ATP Assay in the Field

**Figure 8:**
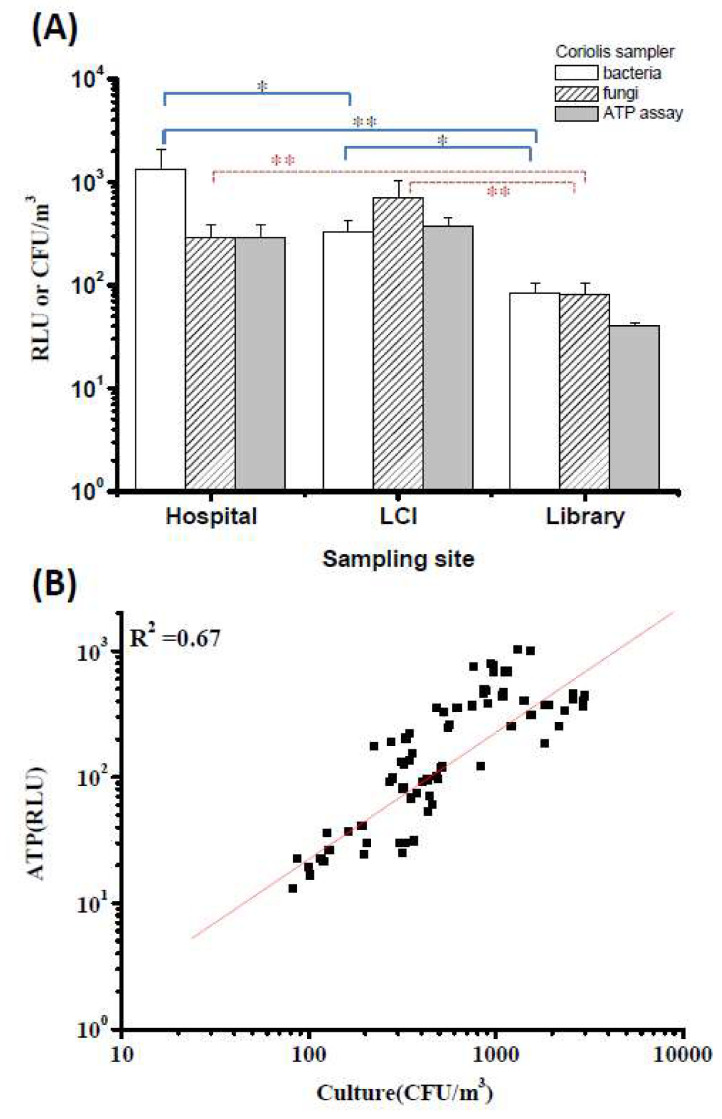
(**A**) Culturable and viable concentrations of bacteria and fungi and their corresponding RLU values in the air of hospital halls, libraries and long-term care institutions (LCIs) detected by a Coriolis sampler. * *p* < 0.05 and ** *p* < 0.01 compared with the respective groups. The experiments were performed in triplicate, and the data are shown as the mean ± standard error of the mean. (**B**) Correlation between total culturable microbes (bacteria + fungi) and their corresponding RLU values detected by the Coriolis sampler.

During the field study periods, we went to two hospital halls, two LCIs, and two libraries for bioaerosol sampling in eastern Taiwan. We obtained a total of 157 samples and determined their airborne bacterial and fungal concentrations and corresponding ATP levels. During the sampling periods, the average temperature ranged from 22.8 °C to 26.7 °C, and the average RH ranged from 67.9% to 74.4% among the three sampling sites (Table S1). Since all of these buildings had mechanical ventilation and air conditioning, there was no significant difference in temperature and RH among the three sampling locations. The mean culturable concentration of total bacteria collected using the Coriolis sampler was 1343 CFU/m^3^ in the hospitals, 331 CFU/m^3^ in the LCIs and 85 CFU/m^3^ in the libraries (Figure 8A). In addition, the average fungal concentrations were 287, 700 and 371 CFU/m^3^ in the hospitals, LCIs and libraries, respectively (Figure 8A). The ATP level was the highest in the LCIs, followed by the hospitals and libraries. Overall, hospital air contained the highest bacterial concentration, and the highest fungal concentration was in the LCI air. Not surprisingly, the library contained the lowest concentrations of all tests, including those determined by ATP assay, exhibiting significant differences compared to the concentrations for hospitals and LCIs (*p* < 0.001). A limitation of the ATP assay is that it cannot distinguish whether the RLU values are obtained from bacteria, fungi or other organisms. Consequently, we summed the concentrations of bacteria and fungi and applied linear regression to investigate the relationship between total culturable (combined bacterial and fungal) concentrations and their corresponding RLU values. There was a significant correlation (*p* < 0.001) and a moderately strong relationship (*R*^2^ = 0.67) between total bacterial/fungal concentrations and RLU values (Figure 8B).

### 3.7. The ATP Criterion Indicates Airborne Bacterial and Fungal Levels by ROC Curves

**Figure 9:**
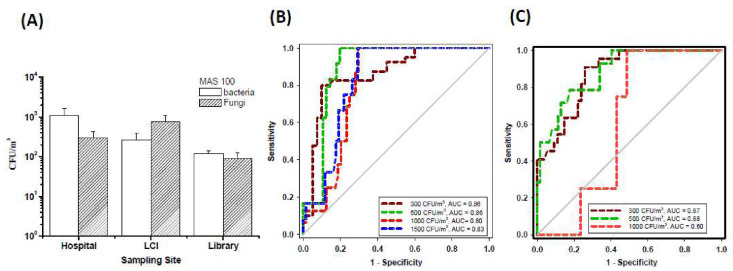
(**A**) Total culturable concentrations of bacteria and fungi in the air of hospital halls, libraries and long-term care institutions (LCIs) detected by a MAS 100 impactor. Receiver operating characteristic (ROC) curves for the criteria were based on results obtained with the MAS 100 sampler. The dotted lines in different colors represent the cases in which (**B**) bacteria and (**C**) fungi culture-based levels of <300 (brown), <500 (green), <1000 (red) and <1500 (blue) CFU/m^3^ were regarded as “qualified” compared with these published guidelines. The best cutoff points demonstrated by all curves were determined using the Youden index and presented in the text. AUC represents the area under the curve.

Since most threshold limits of bioaerosols have been based on impactor collection, the culture-based results in Figure 9A were obtained with a MAS 100 impactor. In general, the results obtained from parallel sampling with the MAS 100 (Figure 9A) were not significantly different from those obtained with the Coriolis sampler (Figure 8A; *p* = 0.17).

ROC curve analysis showed that possible criteria corresponding to the ATP levels determined by the Coriolis sampler could predict the subsequent culture-based results (by the MAS 100) at levels that were above or below several published guidelines. According to the literature, several guideline values for airborne bacterial concentrations could be roughly divided into representative values: 300, 500, 1000 and 1500 CFU/m^3^. All values of the area under the curve (AUC) were higher than 0.80, indicating that the ATP levels determined with the Coriolis sampler provided good discrimination of airborne bacteria for the different levels (Figure 9B; *p* < 0.001). The best cutoff points determined using the Youden index were 258 RLUs for 300 CFU/m^3^ (sen: 80%; spe: 90%), 287 RLUs for both 500 CFU/m^3^ (sen: 100%; spe: 80%) and 1000 CFU/m^3^ (sen: 100%; spe: 70%) and 330 RLUs for 1500 CFU/m^3^ (sen: 100%; spe: 71%). The sensitivity level of the ROC curve analysis indicated the proportion of ATP samples with values that were higher than the corresponding cutoff points and yielded a culturability over the abovementioned guideline values. Additionally, the specificity indicated the proportion of ATP samples with values that were lower than the corresponding cutoff points and yielded a culturability lower than the guideline values.

Similar to the bacterial aerosol guidelines, the three fungal levels 300, 500, and 1000 CFU/m^3^ have often been recommended in office or other indoor environments (Figure 9C). The AUC values were higher than 0.80 for the ROC model for predicting CFU levels higher than 300 and 500 CFU/m^3^ (*p* < 0.001). The best cutoff points of these two ROC models were 251 RLUs for 300 CFU/m^3^ (sen: 91%; spe: 74%) and 370 RLUs for 500 CFU/m^3^ (sen: 79%; spe: 82%). However, the AUC value was only 0.60 when it was estimated whether the CFU levels were higher than 1000 CFU/m^3^ (*p* = 0.49).

## 4. Discussion

The concentration range (10^−3^ to 10^−10^ mM) of the ATP standard curve evaluated in this study was based on those of previous studies [2,16]. Most ATP contents of indoor bioaerosols fall within this concentration range. Taking into account the detection limit, the ATP detection performance of the portable device in our study was nearly 10 times higher than that of another cell viability assay coupled with the use of a desktop luminometer [2]. In addition, the regression coefficient of the standard curve generated with the portable device (*R*^2^ = 0.992) was slightly lower than that generated with the desktop luminometer (*R*^2^ = 0.998) [2]. Although the portable ATP device may not have performed as well as the desktop luminometer, it still provided acceptable ATP detection performance according to our viability test in the chamber and field studies. The advantage of a portable device is its availability and mobility: it can be applied to evaluate high concentrations of bioaerosols in occupational environments or to preliminarily identify potential indoor air quality problems in the field.

Compared with other bioaerosol studies with respect to ATP, our study investigated more microbe varieties, including a yeast and endospores [2,3,13,16]. Most studies have used *Staphylococcus* spp. as a target microbe, but this did not represent the real bioaerosol composition in the field. Fungal spore detection was not conducted in our study because these spores have very limited ATP contents [25]; furthermore, fungal spores were not suitable for aerosolization by the nebulizer because of their hydrophobicity. The ATP concentration at the single culturable cell level in the different microbial species of our study was very consistent with that of a previous study [25]. The order of ATP contents per cell was yeast fungi (*C. famata*; 2.0 × 10^−^^14^ moles) > gram-positive bacteria (*S. aureus*, 2.8 × 10^−^^16^ moles) > gram-negative bacteria (*E. coli*; 4.3 × 10^−^^17^ moles) > endospores (*B. subtilis*; 1.1 × 10^−^^20^ moles). A relatively large number of yeast and gram-positive bacterial cells in the air may contribute to a relatively high ATP content; by contrast, a relatively large number of gram-negative bacteria and endospores may result in a relatively low ATP level. To date, limiting bioaerosol levels to the levels in quantitative standards or guidelines based on microbe culturability in different environments has been recommended [22,23,24,26]. To see if the bioaerosol concentration exceeded these guideline values on site, the relationship between culturable cells and their ATP contents was more important than the number of total cells.

The integrity of the microbial cell membrane is a commonly used indicator for investigating the viability of microbes. Our previous studies used a DNA-binding agent (propidium monoazide) coupled with a real-time quantitative polymerase chain reaction to demonstrate that heating might damage the integrity of the cell membrane and even that DNA can be released from bacterial cells [9,27]. Although a relatively high standard deviation of the endospores in the viability test demonstrated that they were relatively unstable in these samples compared to the cellular samples, the ATP assay indicated that heat stress was sufficient for reducing endospore RLU values and was correlated with their culturability. Therefore, the ATP assay is a good alternative method for analyzing microbe viability.

A Coriolis sampler is a cyclonic instrument that collects particles by fixing them in a liquid. For wet-cyclone sampling, the loss of collection liquid in the cyclone would affect its collection efficiency. The loss of liquid during sampling is related to evaporation, and the most affected parameter is humidity [28]. In fact, in addition to the loss of collection liquid affecting the collection efficiency, excessive supplementation of the liquid might also reduce its efficiency [29]. A sufficient liquid medium volume would enrich the captured particles and maintain the microbe culturability and viability during a long sampling period [30]; cyclonic sampling instruments are usually operated at high sampling flow rates [28]. In addition, a stable volume of the collection liquid is required for the formation of a stable film covering the entire surface of the cyclone to prevent sample loss [29]. Most studies applying the Coriolis sampler for sampling do not discuss the issue of real-time compensation for the loss of liquid, which may affect the sampling efficiency of the instrument. Two previous studies recommended that the compensation liquid flow rate is approximately 0.5 mL/min at an RH of 60% or that it is weather-dependent [28,31]. However, their sampling flow rates ranged widely, from 200 to 630 L/min. Our study demonstrated that even at a short sampling time of 5 min at 200 to 300 L/min, nearly 20% of the liquid was lost. Therefore, in addition to the surrounding RH, the sampling flow rate and time should also be considered to determine the compensated liquid volume when sampling with the Coriolis sampler.

Our study used sterile deionized water as the collection liquid inside the BioSampler and the Coriolis sampler because a previous study indicated that some components in liquid may cause optical interference and decrease ATP levels [2]. The use of water as the collection medium in both samplers may have not maintained the optimal culturability of the microorganisms, but all bioaerosols in our chamber were stable (CV < 10%) and did not interfere with the selection of the optimal sampling conditions. In addition, in the field study, the sampler used for determining whether the concentration of bioaerosols exceeded the standard or guideline levels was the MAS 100 impactor, not the Coriolis sampler. Therefore, the optimal sampling conditions of the Coriolis sampler were primarily determined by the viable efficiency, but the results of culturable efficiency could also be used as a reference.

The BioSampler was used as a reference sampler since it has a high physical collection efficiency of >95% for particle sizes larger than 1.0 μm [32,33]. In addition, it was considered to preserve the culturability of bioaerosols since it minimizes impaction stress during sampling [34]. Compared with the BioSampler, the Coriolis sampler had lower physical collection efficiency, and the efficiency varied from 70% to 90% with particle sizes ranging from 0.5 μm to 1.6 μm [35]. The Coriolis sampler has already been applied for the detection of various biological species. However, most studies address only the biological efficiency regarding the recovery of culturability, not viability. Our study recommended using the Coriolis sampler instead of the BioSampler because of the former’s high sampling flow rate, which may achieve a significantly lower detection limit than traditional samplers, including the BioSampler [35,36]. Although a high sampling flow rate may decrease the viability of bioaerosols [37], our results demonstrated that the mechanical stress caused by impaction and washing steps has a limited effect on the recovered ATP of bioaerosols.

Regarding the culturable efficiency of the two sampling devices, the performance of the BioSampler was found to be better than that of the Coriolis sampler when collecting airborne *S. aureus* and *B. subtilis* endospores. Microbes tangentially impacted on the wall may undergo mechanical stress that affects their culturability, and the corresponding impact is dependent on the stress tolerance of the microbe. However, the culturable efficiencies of the Coriolis sampler under optimal conditions (200 L/min × 30 min) were not significantly different from those of BioSampler for 30 min of sampling. For the Coriolis sampler, our results agreed with those of a previous study, specifically, that gram-negative bacteria are much more susceptible (with a lower CR) to sampling stress than gram-positive bacteria [34], bacterial spores [34] and even fungal cells.

Combining the viable and culturable efficiencies for the Coriolis sampler shows that the recovery rates in terms of the viable efficiencies of all four types of microbes were stable and much higher than the recovery rates in terms of culturable efficiencies. This phenomenon explains why microbial cells containing ATP can be successfully recovered by the Coriolis sampler, but the culturability of these microbes may be species dependent and their culturability could be individually affected by varying sampling conditions. Therefore, some microbes may remain in a viable but not culturable state after sampling [38]. The optimal conditions for the Coriolis sampler were based on considering the highest viable and culturable efficiencies of the microbes collected with the Coriolis sampler and were then determined statistically with a post hoc comparison. In comparison with our optimal conditions, the frequently used conditions (300 L/min for 5 or 10 min) for the Coriolis sampler applied in previous studies [35,39] were similar in terms of viable efficiency, but the culturable efficiency could be lower than that obtained with our optimal conditions.

Within the field sampling period, there were twice as many or even more people in the hospital than in the LCI or library, which may account for the greater concentration of total bacteria observed in the hospital. Moreover, since the LCIs have more open space than the other sites and have green plants outside, the fungal concentrations were higher there than at the hospitals and libraries. The total bacterial concentration (1000–1500 CFU/m^3^) from previous sampling at the same hospital was similar to that of our current study [27]. The total bioaerosol concentration in the hospital hall was 3.7 times higher than that in the ward, and the total concentration in the LCIs was 2.0 times higher than that in a health center [40]. For the LCI and library sampling, there was a less than 2-fold difference in bioaerosol concentration compared to those of previous studies [41,42]. These results indicated that the bioaerosol concentrations measured in this study were close to those at the similar sampling sites investigated in previous studies. However, some sampling locations with large differences, such as hospital halls and wards or LCIs and health centers, have greater differences in bioaerosol concentrations. Linear regression results showed that there was a significant correlation between ATP counts and total colony counts: 1 RLU corresponded to nearly 0.1 CFU (Figure 8B). Venkateswaran et al. hypothesized that the predominant type of microbe in samples can be indicated by the ratio of RLU to CFU [25]. A low RLU:CFU ratio, as presented in our study, is expected to indicate bacterial dominance, and the overall culturable concentration of bacteria was indeed higher than that of fungi. Conversely, if samples were fungus dominant, the RLU:CFU ratio would have been relatively high.

The major purpose of our study was to identify an “acceptable cleanliness” cutoff for the ATP level; it could be helpful for predicting whether the determined bioaerosol concentration presented in CFU/m^3^ on site is acceptable or not. At present, the maximum allowed values for total bacterial aerosols have been legislated in Korea (800 CFU/m^3^) [43] and Taiwan (1500 CFU/m^3^) [44]. In addition, Hong Kong also recommends that a total bacterial aerosol level of 500 CFU/m^3^ is “excellent” for indoor air quality and that 1000 CFU/m^3^ is in the “good class” [45]. In comparison with those for total bacteria, the proposed criteria for fungal aerosols varied widely in different environments [22,23,24,26]. Although the evidence to prove the relationships between the exposure dose of bioaerosols and related adverse health effects is still insufficient, recommendations or guidelines are still valuable for facilitating the interpretation of measured values [46].

Our current study used the MAS 100 impactor instead of the Coriolis sampler to obtain culturable data since most recommended protocols for measuring bioaerosols utilize different types of impactors [22,23,24]. When using the obtained ATP levels to predict the CFU/m^3^ values on site, most studies applied a linear regression model. However, this approach is difficult to use since the ATP assay cannot distinguish microbial strains and the composition of bioaerosols in the field is diverse. Therefore, it may be more feasible to use the ROC model to predict whether the total accumulated ATP level exceeds a certain standard. In addition, the results of the ROC model also demonstrate its sensitivity and specificity; these two indicators allow people to understand the credibility of the predicted value. In fact, several benchmarks in terms of the RLU values have already been established to indicate whether the surface cleanness could be successfully “qualified” by the ROC model [17]. However, this kind of approach has not been applied to bioaerosol investigations.

According to the established ROC model, it was possible to predict whether the culturable concentration of airborne bacteria or fungi exceeded varied standards through using different optimal cutoff points. Our study demonstrated that the cutoff points used for determining whether the concentration of culturable bacteria exceeded 500 or 1000 CFU/m^3^ were the same. Based on the relatively high AUC value and sufficient sensitivity and specificity, we recommend using the value of 257 RLUs for predicting whether the concentration exceeds 500 CFU/m^3^. For fungal aerosols, the ROC model used to predict whether the culturable concentration of fungal aerosols is over 1000 CFU/m^3^ did not perform well. This may have been related to only 5% of the environmental fungal samples having a concentration above 1000 CFU/m^3^. More indoor environments with high concentrations of fungal aerosols must be sampled to improve the predictive effect of this ROC model.

## 5. Conclusions

Rapid bioaerosol monitoring is important for controlling indoor air quality. This study shows that both culturable and viable efficiencies of the Coriolis sampler under optimal conditions (200 L/min × 30 min) were not significantly different from those of sampling by BioSampler for 30 min. In the field, several cutoff RLU values were identified by the ROC model to indicate whether the culturable bioaerosol concentration on site could be higher or lower than the different established guideline or regulation values. Our study recommended that the Coriolis sampler coupled with a portable ATP bioluminescence assay is an available, affordable and quick analytical method. The recommended approach in this study can produce bioaerosol results in a short time (< 1 min). However, if the established ROC model is required to be applied to indoor environments that are quite different from hospital halls, LCIs and libraries, additional field tests may need to be conducted.

## Supplementary Materials

Supplementary materials can be found at https://www.mdpi.com/2076-2607/8/7/975/s1. Table S1. Temperature and relative humidity of the three sampling sites.
