# Supplementary material for: Optimization of a Portable Adenosine Triphosphate Bioluminescence Assay Coupled with a Receiver Operating Characteristic Model to Assess Bioaerosol Concentrations on Site
Source: Microorganisms. 2020 Jun 29;8(7):975. doi: 10.3390/microorganisms8070975 (PMC7409044; doi:10.3390/microorganisms8070975)
Supplement: Supplementary file 1 [file microorganisms-08-00975-s001.pdf]

Table S1. Temperature and relative humidity of the three sampling sites.

	Hospitals	Long-Term Care Institutions	Libraries
Temperature (°C)	22.8 (4.7)	26.7 (1.8)	23.0 (1.1)
Relative humidity (%)	73.6 (4.6)	74.4 (5.3)	67.9 (3.5)

The numbers in parentheses represent standard deviations.
